# Comparative epidemiology of poliovirus transmission

**DOI:** 10.1038/s41598-017-17749-5

**Published:** 2017-12-12

**Authors:** Navideh Noori, John M. Drake, Pejman Rohani

**Affiliations:** 1 Odum School of Ecology, University of Georgia, Athens, GA USA; 2 Center for the Ecology of Infectious Diseases, University of Georgia, Athens, GA USA; 3 Department of Infectious Diseases, University of Georgia, Athens, GA USA

## Abstract

Understanding the determinants of polio transmission and its large-scale epidemiology remains a public health priority. Despite a 99% reduction in annual wild poliovirus (WPV) cases since 1988, tackling the last 1% has proven difficult. We identified key covariates of geographical variation in polio transmission patterns by relating country-specific annual disease incidence to demographic, socio-economic and environmental factors. We assessed the relative contributions of these variables to the performance of computer-generated models for predicting polio transmission. We also examined the effect of spatial coupling on the polio extinction frequency in islands relative to larger land masses. Access to sanitation, population density, forest cover and routine vaccination coverage were the strongest predictors of polio incidence, however their relative effect sizes were inconsistent geographically. The effect of climate variables on polio incidence was negligible, indicating that a climate effect is not identifiable at the annual scale, suggesting a role for climate in shaping the transmission seasonality rather than intensity. We found polio fadeout frequency to depend on both population size and demography, which should therefore be considered in policies aimed at extinction. Our comparative epidemiological approach highlights the heterogeneity among polio transmission determinants. Recognition of this variation is important for the maintenance of population immunity in a post-polio era.

## Introduction

During the late nineteenth and early twentieth centuries, poliomyelitis, an acute viral disease, caused the paralysis of hundreds of thousands of children^1^. The introduction and widespread use of effective vaccines in the 1950 s and 1960 s led to a dramatic reduction in polio-related Acute Flaccid Paralysis (AFP). Since the launch of the Global Polio Eradication Initiative (GPEI) in 1988, more than 2.5 billion children have been vaccinated with a corresponding decline of over 99% in the annual wild poliovirus (WPV) incidence^2^. Despite this impressive progress, tackling the last one percent of polio cases has been challenging^3^. In addition to the failure to reach pockets of unvaccinated children due to conflict, religious beliefs and social upheaval^4^, other factors might help virus circulate in the environment^5^, but their effects are poorly understood. The large scale geographic variation in incidence and frequent historical spillover of WPV from regional strongholds to countries believed to be polio-free highlight the urgent need to identify local conditions conducive to ongoing transmission^6^. An understanding of the covariates of poliovirus incidence is also important for risk management in countries that have been declared disease-free.

Human infectious diseases display striking biogeographic patterns at a global scale^7^, which may often be explained by demographic, environmental, and socioeconomic factors. Often, these patterns contain the clues needed to identify key risk factors. Assessing the variation in the magnitude of disease outbreaks and their geographic consistency with respect to these factors–an approach referred to as *spatial epidemiology* ^8^–is necessary for characterizing infectious disease dynamics at the global scale^5,9,10^. In recent years, spatial epidemiology has been used to explore the dynamics of SARS^11^, whooping cough^12^, foot-and-mouth disease^13^, rotavirus^14^, pandemic influenza^15^, and polio^16^. Studies of polio, however, have focused almost exclusively on vaccination policies, using either predictive statistical models^17–19^ or dynamic models of transmission^20,21^. A quantified picture of regional disease patterns and their spatially extended macroecological determinants is still missing. To bridge this gap, we performed a comparative study, investigating a wide range of potential predictors of polio incidence at the national level. We were particularly interested in identifying the drivers of poliovirus persistence in the environment. Our results show that there is not a singular “polio epidemiology” or social syndrome hampering elimination, but rather that the conditions supporting persistence vary from place to place and require local knowledge to identify and overcome.

Our study was guided by patterns discovered in the data during analysis. Statistical models were used to compare covariates from across demographic, social, and environmental categories of putative causation. First, we studied factors known to drive the replenishment rate of the population susceptible pool. These included vaccine uptake and demographic factors such as the *per capita* birth rate and population density^22^. Second, we considered potential feedbacks between health, infectious disease transmission and poverty^23^ by quantifying the relationship between polio incidence and *per capita* Gross Domestic Product (GDP). Since the polio virus is transmitted via the fecal-oral route, we supposed that exposure of infectious virus particles to external environmental conditions may be important. Thus, we also considered factors conjectured to affect viral persistence and exposure risk, particularly changes in land use, climate, and indicators of sanitation and hygiene^24,25^. By grouping variables in this way, we consider how the overall dynamical response of polio to interventions taken to eliminate the last $1 \%$ may differ from those in place during the past four decades and in countries currently designated as polio-free.

Permanent disease elimination depends on both stopping local transmission and preventing re-introduction. Thus, in addition to analyses that focus on *local* determinants of incidence, we also evaluated polio *regional* persistence. Specifically, we examined the Critical Community Size (CCS)^26^, which is the theoretical threshold population size above which an infectious disease persists strictly through local transmission^27–29^. In practice, it has been found that the realized CCS is determined by a combination of the susceptible influx rate^26^ and mobility among communities^30^. To assess the effect of spatial coupling on the extinction rate, we compared the extinction profile of polio in non-island and island countries. Together, these analyses document the effects of a combination of factors, including socio-economic, demographic and environmental conditions, on the frequency and amplitude of polio incidence. We highlight the conditions that might be favorable for poliovirus transmission. This is crucial especially in the absence of detected AFP cases and the silent circulation of poliovirus. This work paints a global picture of the changes in polio incidence over the past four decades. It reveals a virus whose transmission dynamics and regional persistence defy a simple explanation and are instead determined by an ensemble of factors.

## Results

### Polio Incidence

**Figure 1:**
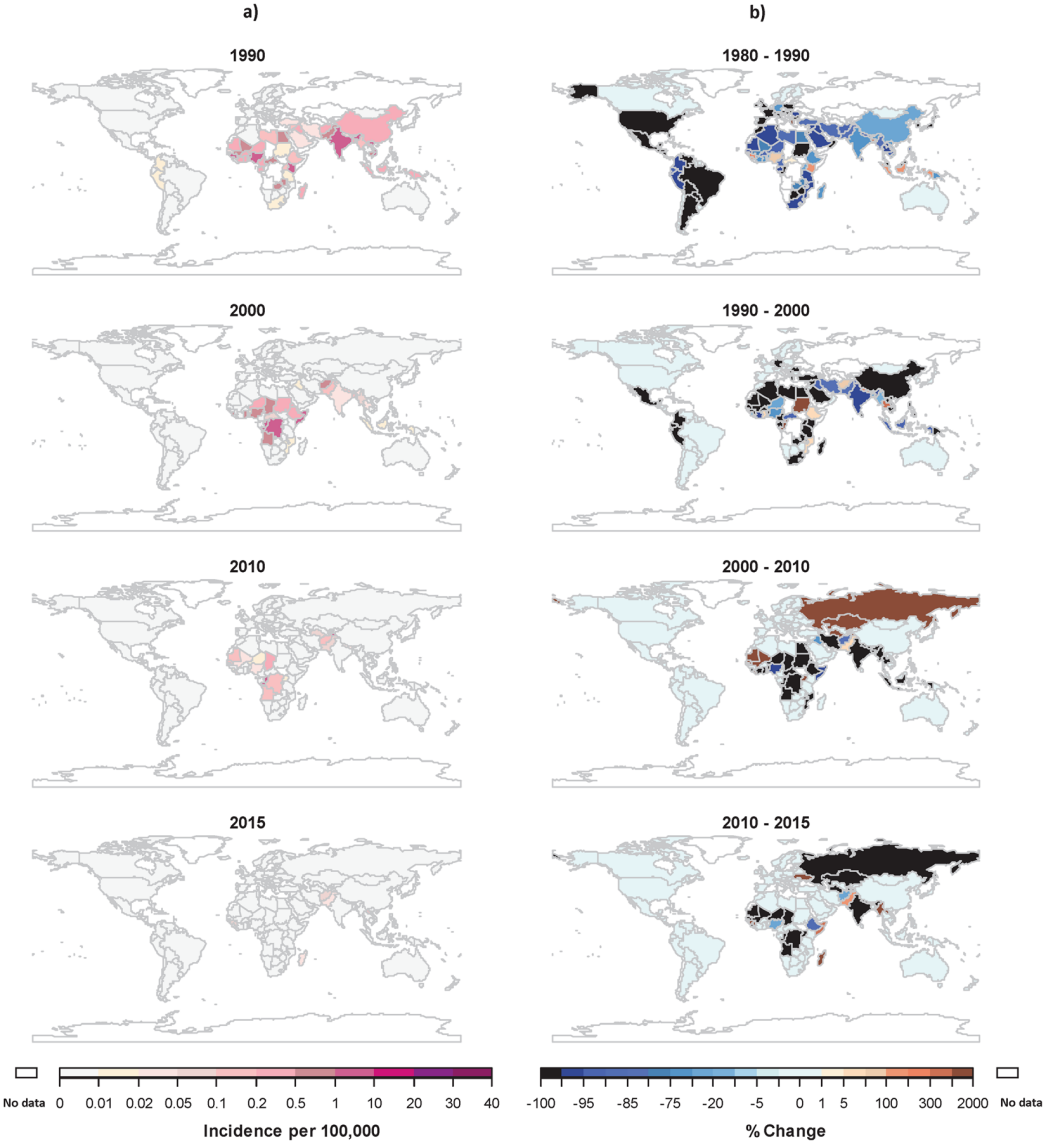
(**a**) Incidence per 100,000 for each country for the years 1990, 2000, 2010 and 2015. (**b**) Percent changes in incidence of polio per country for the periods 1980–1990, 1990–2000, 2000–2010 and 2010–2015. Black color indicates zero incidence at the end of each period. (R Core Team (2016)^71^).

Historical polio incidence maps highlight a major reduction in the geographic range of polio (Fig. 1(a)). By 2015, polio incidence had been restricted to a handful of African nations, together with Pakistan and Afghanistan. These maps also point to substantial epidemiological heterogeneity across countries. Despite the overall declining trend (Fig. 1(b)), numerous spatially localized outbreaks are evident (Fig. 1(a)). For instance, polio incidence dropped substantially in Madagascar from 1990 to 2000, but was followed by an outbreak in 2015. These maps also depict the increasing number of polio-free countries through time (shaded light blue in Fig. 1(b)).

**Figure 2:**
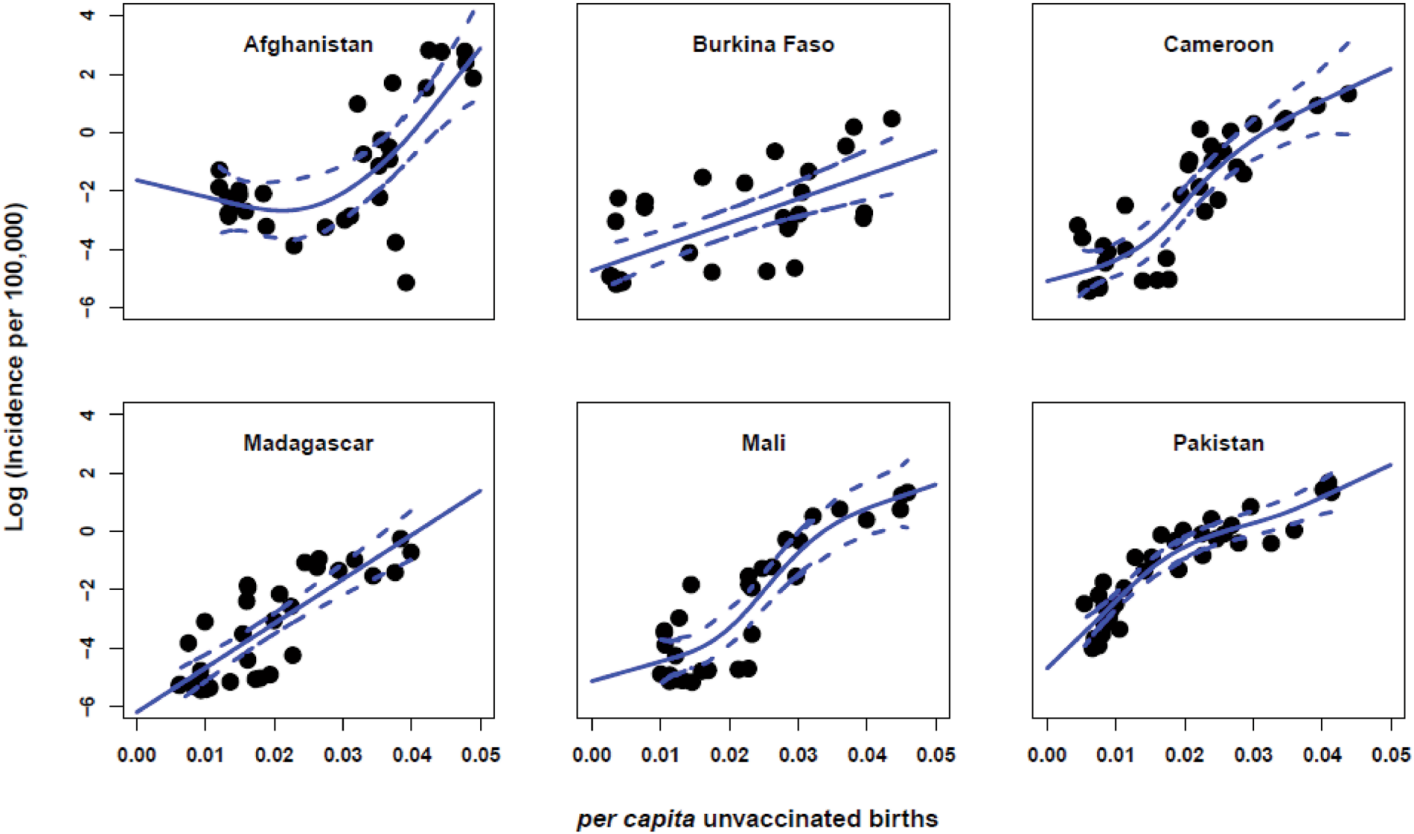
Plots of logarithmic incidence rate against *per capita* unvaccinated births for six different countries. Generalized additive model (GAM) was fitted to each country data points. Dash lines shows the confidence interval of the GAM model.

As predicted by epidemiological theory^31,32^, we observed a strong association between polio incidence and the rate of unvaccinated births (Fig. 2). In particular, increased vaccination coverage (which reduces the influx of unvaccinated births) was associated with declines in polio incidence. In some countries, such as Burkina Faso, Mali and Afghanistan, a threshold in unvaccinated births was identified by segmented regression models, below which there was little change in polio incidence (see Supplementary Fig. S5). These findings highlight the expected combined effects of vaccination coverage and population demography in modulating population-level poliovirus circulation. We note, however, that a threshold in unvaccinated births was not detected in all countries, suggesting that the variation in country-specific incidence cannot be explained by unvaccinated births alone. There must therefore be additional factors underlying this heterogeneity.

**Figure 3:**
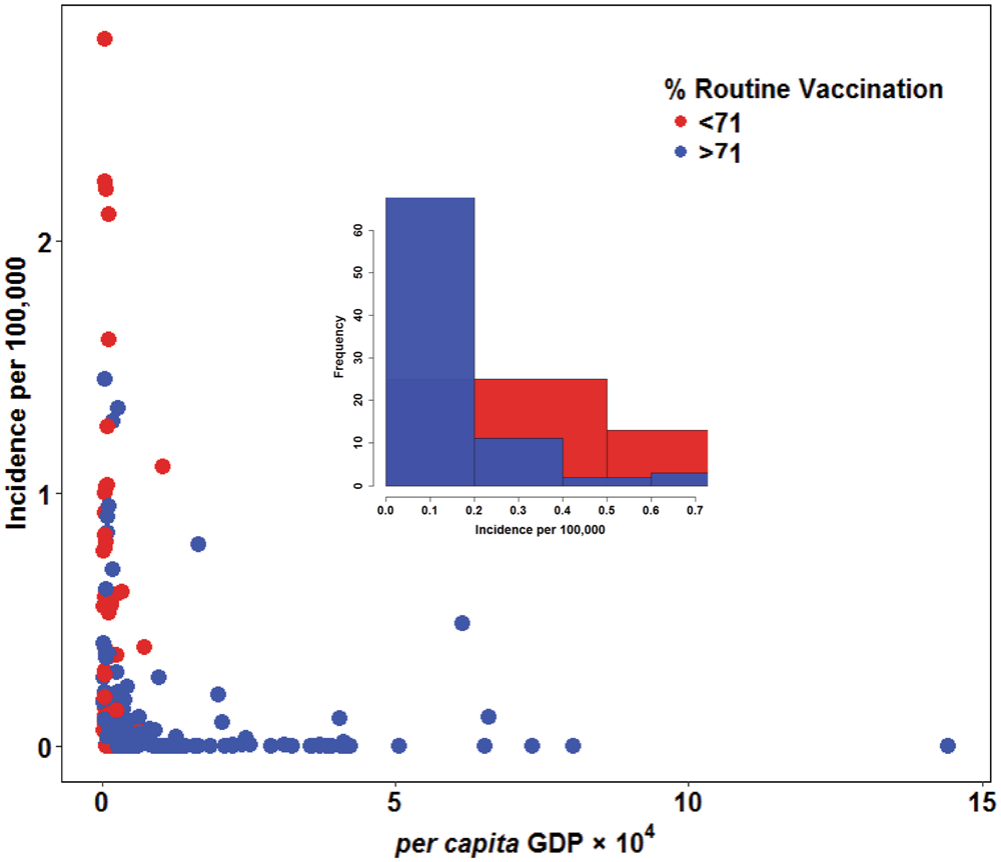
Polio Incidence per 100,000 and *per capita* GDP (constant 2010 US$) averaged over the period 1980–2015. Each point represents a country. Blue color points indicate countries with the vaccination coverage >71% and red color points indicate countries with the vaccination coverage <71%.

To explain this heterogeneity, we first explored the impact of economic development on polio by plotting country-specific annual *per capita* GDP against polio incidence (Fig. 3). Strikingly, the data form an L-shape, aligning with either the x- or the y-axis, indicating a strong association between *per capita* GDP and polio incidence. Essentially, countries whose GDP exceeds $$\$\mathrm{1,000}$$ per person per year are polio free, with a significant reduction in model deviance using the smoothed response to vaccination (P-value = 0.002). There was also a significant smoothed response to the interaction between GDP and vaccine uptake for countries with *per capita* GDP > $$\$\mathrm{1,000}$$ (P-value = 0.022). Among countries whose GDP is less than $1000, the smoothed response to vaccination was statistically significant (P-value = 0.048) (see Supplementary Table S1 and Fig. S6). Lower income countries carry the overwhelming majority of the disease burden. To further dissect the impact of *per capita* GDP and vaccination coverage on polio incidence, a GAM was fitted to polio incidence against GDP. A subsequent ANOVA performed on the residuals of the fitted model showed that the variation in polio incidence that could not be explained by *per capita* GDP, is significantly related to vaccination (P-value < 0.001), providing evidence of a combined effect of income and vaccine uptake (Table 1).

**Table 1:** Analysis of Variance performed on residuals of fitted GAM to polio incidence and *per capita* GDP, versus vaccination coverage.

	df	F value	p-value
Vaccination	1	17.13	<0.0001

### Ranking the Determinants of Polio Incidence

For random forests models, we ranked each country according to the predicted impact of potential covariates of polio transmission based on $\Delta$ MSE. The ${R}^{2}$ of fitted random forests models across countries ranged from 0.74 to 0.98 (see Supplementary Table S3). Population density, percent of people with access to improved sanitation facilities, and percent forest cover were ranked as the top three most important variables among 64%, 54% and 43% of countries (Table 2). For only 29% of countries, *per capita* unvaccinated births and percent urban population growth were also identified as significant correlates of polio incidence. Climatic variables were consistently among the least important predictors, irrespective of region. The single random forests model fitted to countries all-together, also showed similar results to those for country-specific fits. However the percentage of variance explained by the single model was only 21% (see Supplementary Fig. S7 and Table S2).

**Table 2:** Percent countries having each predictor as their top 3 important parameters among regions and within each region based on the random forests model.

Variables	All Regions	African	Eastern Mediterranean	South-East Asia	Western Pacific	Americas	Europe
% of people with access to improved sanitation facilities	0.54	0.53	0.31	0.57	0.67	0.83	0.67
% Forest land	0.43	0.53	0.15	0.14	0.33	0.83	0.67
% Arable land	0.2	0.18	0.31	0.43	0	0	0.33
*per capita* GDP	0.25	0.24	0.31	0.29	0.33	0	0.33
Population density	0.64	0.71	0.62	0.43	0.5	0.67	0.67
% Rural population growth	0.26	0.26	0.23	0.57	0.33	0	0
% Urban population growth	0.29	0.21	0.46	0.14	0.5	0.33	0.33
Average temperature	0.06	0.09	0.08	0	0	0	0
Total precipitation	0.04	0.06	0.08	0	0	0	0
*per capita* unvaccinated births	0.29	0.21	0.46	0.43	0.33	0.33	0

In analyses such as this, it is possible for association among predictors to mask the true relationship between underlying covariates and response. To identify any possible grouping among predictors, we calculated the correlation matrix of the rankings of predictors, estimated by the random forests models. We failed to detect any systematic relationship in the predictive ability of covariates globally (See Supplementary Materials and Fig. S8), indicating that there are many statistically significant covariates of change in polio incidence rather than a universal pattern.

**Figure 4:**
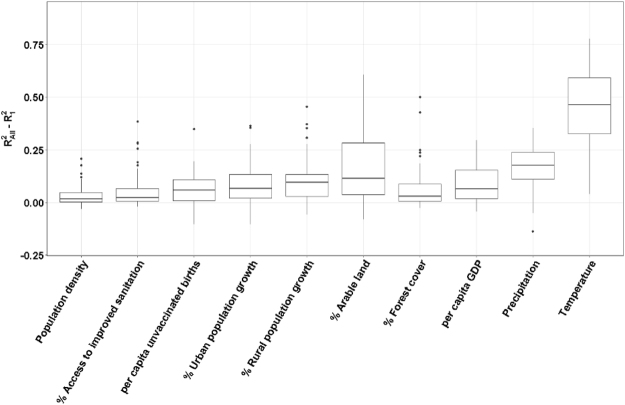
Difference in R^2^ of random forest models developed using (1) all predictors and (2) only one predictor.

We also set out to further quantify the relative importance of each covariate to the variation in polio incidence. Particularly, we contrasted the ${R}^{2}$ of random forests models fitted to all predictors (${R}_{{All}}^{2}$) to that of models in which predictors were fitted one at a time (denoted by ${R}_{i}^{2}$, with $i$ representing a specific predictor). A small value of ${R}_{{All}}^{2}-{R}_{i}^{2}$ is interpreted as indicating a high explanatory value for factor $i$ (Fig. 4). Consistent with the results summarized in Table 2, we found most of the variation in polio incidence to be explained by population density, percent of people with access to improved sanitation facilities and percent forest cover.

### Model Validation and Polio Forecasting

**Figure 5:**
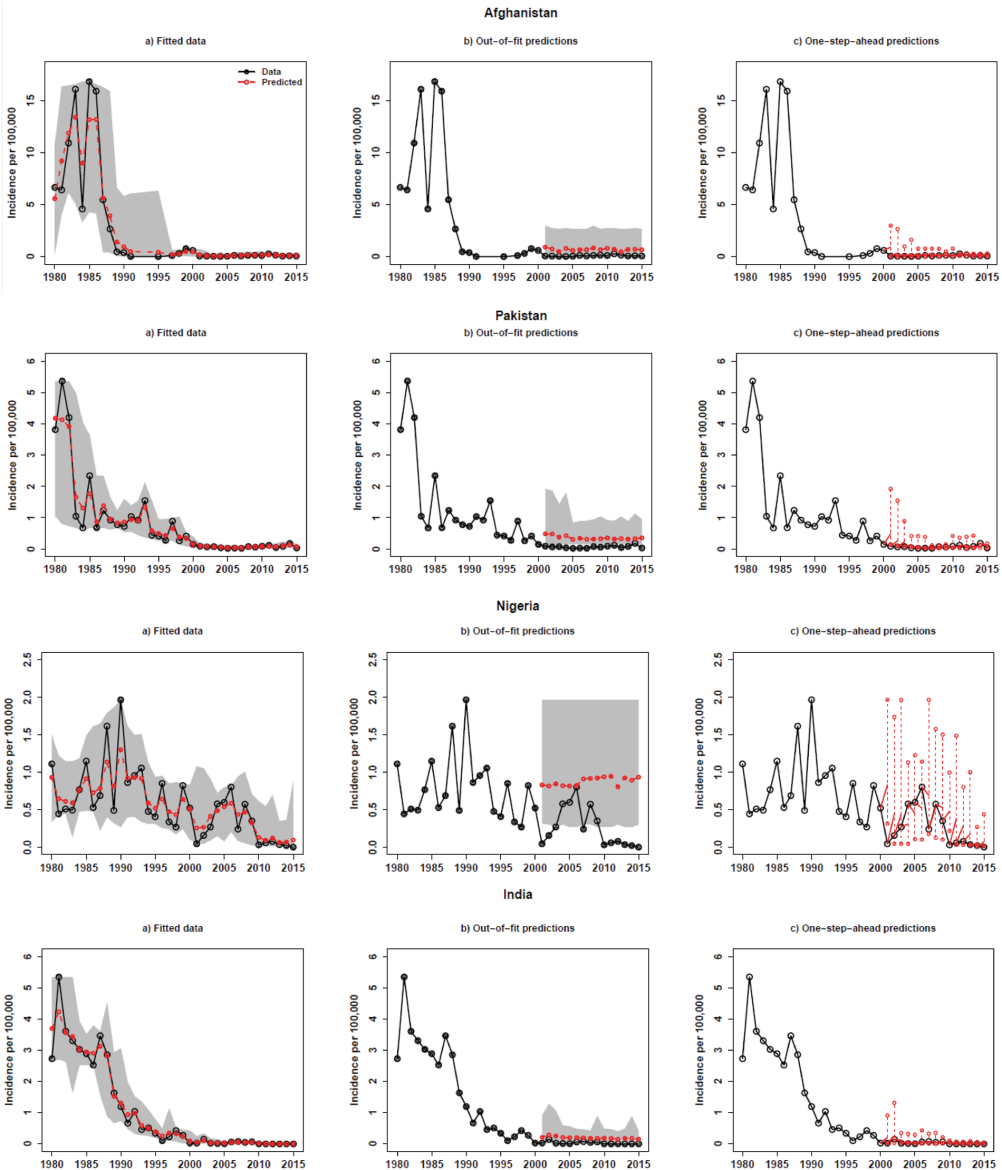
Comparison of predicted polio incidence rate by random forest method versus observed values for Afghanistan, Pakistan, Nigeria and India using three training and testing sets (**a**) fitted data, (**b**) Out-of-fit predictions, (**c**) One-step-ahead predictions.

We evaluated model-data agreement in the random forests models using Afghanistan, Pakistan, Nigeria and India. In general, this exercise showed that, for the fitted data, the random forests model is able to capture key temporal trends in polio incidence, with coefficients of determination of *R* ^2^ = 0.92, 0.95, 0.86 and 0.96, respectively (Fig. 5). We also examined the out-of-sample predictive ability of our fitting procedure. For all four countries, the out-of-fit predictive accuracy of random forests models was low, with wide prediction intervals and small *R* ^2^ values of 0.003, 0.001, 0.16 and 0.44, respectively (Fig. 5). For one-step-ahead predictions, as the number of training points increased, the prediction accuracy improved, with narrower prediction intervals. Finally, to examine the reliability of our modeling approach as a forecasting algorithm, we contrasted its performance against linear regression models (see Supplementary Fig. S9). For the fitted data, random forests models better captured the fluctuations in the data (linear regression: *R* ^2^ = 0.74, 0.78, 0.33 and 0.91, respectively for Afghanistan, Pakistan, Nigeria and India). For the out-of-fit and one-step-ahead predictions, linear regression models also performed poorly (see Supplementary Fig. S9). We suspect that the poor predictive performance of these models stems from their simplicity and linearity–their structure does not permit an accurate bookkeeping of the size of the susceptible population, the inherent nonlinearity in transmission, and interactions with predictors.

### Polio Persistence

**Figure 6:**
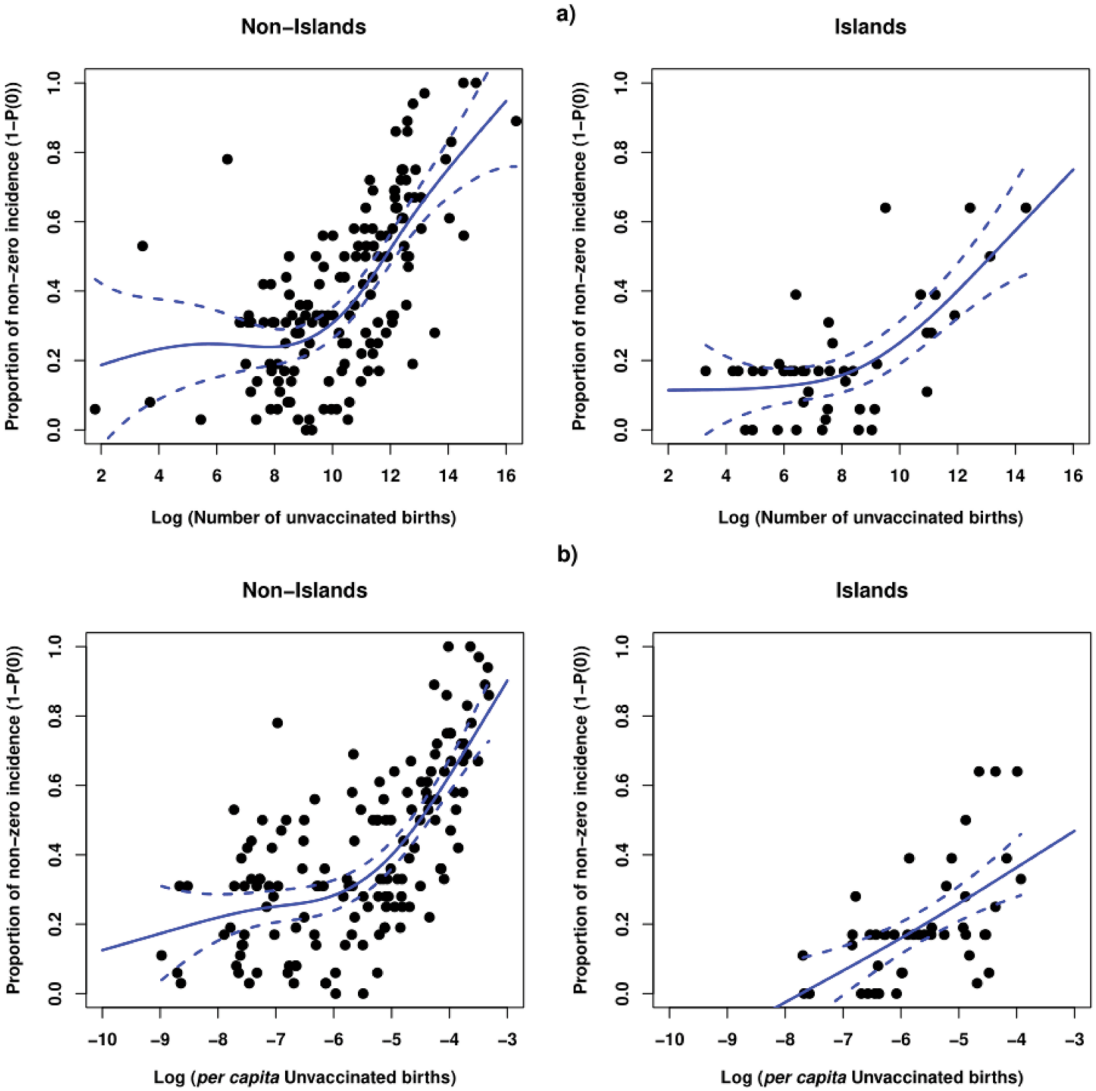
Persistence of polio in island and non-island countries. Y axis is 1- proportion of years in which no cases of polio were reported for the period 1980–2015. X axis is (**a**) number of unvaccinated births and (**b**) *per capita* unvaccinated births. Generalized additive model (GAM) with integrated smoothness estimation was fitted to the data points. Dash lines shows the confidence interval of the fitted model.

Analysis of the regional persistence of polio as a function of the actual number of unvaccinated births for island and non-island countries showed that as the number of unvaccinated births increased, the rate of susceptible replenishment (and, consequently, the probability of disease persistence) increased in both island (*R* ^2^ = 0.53) and non-island (*R* ^2^ = 0.46) communities (Fig. 6(a)). Thresholds in unvaccinated births in both islands (*e* ^8.64±1.62 (s.e.m)^ ≈ 5653, 1118–28566, 95% C. L. and non-islands (*e* ^10.13±0.83 (s.e.m)^ ≈ (25084, 10938–57526, 95% C.I.) were estimated (see Supplementary Fig. S10(a), fitted segmented regression models). Below these breakpoints, polio incidence is unable to remain endemic in the community. ANOVA test showed that the poliovirus fadeout frequency was significantly higher in islands (where CCS is estimated to be smaller) compared to non-islands (P-value < 0.001).

We additionally assessed the role of population size on polio persistence. A plot of the proportion of years with no reported cases of polio versus *per capita* unvaccinated births showed similar results as those shown for the actual number of unvaccinated births especially for non-islands (Fig. 6(b)). The segmented regression model also showed a sharp increase in the relationship between polio persistence and population size, with a more clear breakpoint for non-islands (see Supplementary Fig. S10(b)). This suggests that country-specific polio vaccine coverage targets need to take into account both population size and birth rate to promote local elimination^9^.

## Discussion

This study sheds light on the global epidemiology of polio. The regional heterogeneity in disease incidence is described by a combination of demographic, socio-economic and environmental factors. Socio-economically, we identified a significant interaction between income and vaccine uptake in relation to polio incidence variance. There is a disproportionate burden of infectious diseases on poor nations^23^, whose poverty has, in turn, stymied efforts to interrupt polio virus transmission^33^. In addition to the obvious impact of poverty on health infrastructure and access to vaccines, there is a high proportion of the unvaccinated settlements due to vaccine refusals and missed children^34^ or due to violence and fear caused by the insurgency in endemic countries^35^. Among these countries and other low income countries, it also has been shown that oral poliovirus vaccines (OPVs) exhibit lower immunogenicity compared with high income countries^36,37^. This has presented a major challenge to the global eradication of polio. In low income settings, uncontrolled urban growth may also accelerate overcrowding and lead to inadequate waste-water management, sanitation and public health provisioning, living conditions that are conducive polio virus transmission^38^. Polio cases have been found in both rural and urban settings. Higher incidence in urban areas may arise from overcrowding^39^, while in urban-rural populations it may be additionally due to lack of hygiene and health service facilities^40^.

Our country-specific random forests analysis explained the variation in disease incidence mainly by differences in population density, percent of people with access to sanitation facilities, percent forest cover, and *per capita* unvaccinated births (Table 2 and Fig. 4). We obtained similar results even after fitting a single random forests model to all countries combined as a check against possible idiosyncrasies of this approach (see Supplementary Fig. S7). The importance level of *per capita* GDP for the single random forests model was higher than percent forest cover. However the random forests model fitted to each country separately explained the variance in the data better than the single model (See Supplementary Table S2 and Table S3). Surprisingly, percent forest cover had a positive association with polio incidence (see Supplementary Fig. S11). Forest cover was also negatively correlated with the percentage of people with access to improved sanitation and population density. Accounting for these linkages, we speculate that a higher percent of forest cover in low income countries indicates lower urban development, which may lead to more fragmented public health infrastructure hindering attempts at controlling poliovirus circulation. In addition, we suggest that low percentage of arable land and constrained agricultural growth in most of these poor countries (see Supplementary Fig. S11) may be due to shortage of water supplies and poor agricultural infrastructure. Such limitations can lead to food shortage and malnutrition, which weakens the population resistance to an infection, given exposure^38^. Prevalence of reported cases of polio is higher among vaccinated children with high levels of malnutrition. Poor immunity from malnutrition could lower the efficacy of OPVs and consequently promote vaccine-derived polio outbreaks^41,42^. Also in addition to malnutrition, the oral vaccine failure can be related to shorter duration of breast-feeding in early childhood^43^.

Unexpectedly, we found climate variables contribute very modestly to the predictive performance of our models. This was somewhat surprising given previous work on polio^44^ and other viruses^45,46^ that indicates a significant role for environmental conditions on virus survival and host immunity^47,48^. In particular, a recent study of historic polio trends in the United States identified seasonal transmission peaks organized by latitude^48^, which also suggests a role for abiotic factors in transmission ecology. Our findings do not rule out an effect of climate on transmission, but they do suggest any effects may potentially be more prominent in shaping transmission seasonality^49^ than transmission intensity.

Regarding polio persistence, we found, as expected, extinction frequency to depend on both population size and susceptible recruitment rates: poliovirus persistence is favored in nations that are populous, have high birth rates or low vaccine uptake. Additionally, however, our study also reveals that polio persistence is affected by geography. In particular, higher persistence is observed in non-island communities compared to islands, consistent with other infectious diseases^50^. This may be due to lower spatial coupling in isolated (island) communities leading to less frequent epidemic recolonization and higher overall rates of regional extinctions, compared with non-island populations. Therefore, for the same population size and unvaccinated birth rate, island populations experience higher rates of polio extinction^51^. Thus, long-term polio persistence results from the combined effects of geographic isolation, population demography and vaccine coverage. Especially vaccination affects the structure of disease fade-out. Due to lack of data, we did not consider SIAs in our analyses. In general, higher SIA coverage would be expected to reduce polio persistence.

Our conclusions are subject to number of important qualifications. First, we have implicitly assumed reporting to be consistent through time and we have ignored possible geographic variation in vaccination coverage and disease surveillance both in country and subnational levels^52,53^. Under-reporting of disease incidence leads to an over-estimation of polio extinction frequency, especially in countries with inadequate public health provision and weak surveillance systems. Poor countries with large unvaccinated birth cohorts will report too frequent absence of disease. These limitations induce biases in our analysis. Additionally, for detailed bookkeeping of the susceptible population size, it would be essential to consider additional parallel data on supplementary immunization activity (SIA)^54,55^, vaccine type and composition. Quantification of these factors would serve to improve the association between vaccine coverage and incidence across countries. Also, as previously demonstrated^56^, an assessment of the vaccination history of children not vaccinated during SIAs would help identify the possible pockets of susceptible individuals. In addition, accounting for the geographic variation in quality of conducted SIAs could help describe the heterogeneity in incidence data. Undoubtedly, access to better resolved auxiliary data would help clarify the ecology and the complexity of this important disease system. Although the accuracy of model predictions is limited by the available data, we submit that our analyses on the determinants of polio disease spread and maintenance can give insights into the design of immunization campaigns.

In conclusion, this study paints a global picture of the epidemiology of polio, drawing on the past four decades. Our study indicates that in addition to the poor immunization status, polio persistence resulted mainly from the convergence of environmental, demographic, and socio-economic factors. Addressing the linkage between each of these drivers and polio incidence is necessary to prevent future outbreaks by controlling the polio transmission in a region and also by increasing the efficacy of vaccines. Unbroken chains of poliovirus transmission in any country pose a threat to all countries. There are many small areas within endemic countries where the virus is still present and where surveillance is weak. By linking the knowledge of these areas and their demographic and environmental characteristics to the strategic solutions, the Polio Programme will succeed^34^. In addition, the importance of social data in driving improved performance of the Polio Programme and sound decision making has been frequently emphasized by Independent monitoring board of GPEI^34^. Determination of dominant covariates of polio incidence, would be one step forward towards selection of the right information to make proper decisions. Accordingly, it is expected that specific cases would benefit from the effort required to develop a refined country-specific model accounting for dominant covariates of polio incidence. In addition, recognizing the spatial and temporal incidence patterns has considerable power to help unmask transmission dynamics. In particular, our large-scale comparative approach demonstrated the complexity and heterogeneity among the determinants of polio transmission, shedding light on the intricacies of transmission and the importance of a holistic perspective for effective disease control.

## Methods

### Polio Incidence

We obtained annual polio incidence data from the World Health Organization (WHO) database of country-specific time series of clinical polio cases and estimates of routine vaccination coverage between 1980 and 2015^57^. Incidence data refer to all polio cases (indigenous or imported), including polio cases caused by vaccine derived polio viruses (VDPV). The vaccine data are the WHO/UNICEF Estimates of National Immunization Coverage (WUENIC) and refer to the third dose of polio vaccine - either oral polio vaccine or inactivated polio vaccine (Pol3). Unfortunately, this database does not provide information on supplementary immunization activity (SIA) vaccine type and number of components. Annual *per capita* birth rate and population size were obtained from the World Bank’s World Development Indicators^58^.

To explore the association between incidence, birth rate and vaccination coverage in each country, we fitted a generalized additive model (GAM) with integrated smoothness estimation to the incidence (response variable: number of reported cases with in a year/population $\times$ 100,000) and the susceptible birth rate (predictor variable: (1-vaccination coverage) $\times$ *per capita* birth rate) using the ‘mgcv’ package in R^59^. In additive modeling techniques, the impact of the predictive variables is captured through smooth functions. GAMs can estimate nonlinear relationships between the response variable and the predictor variables without assumptions^60^. The smooth terms for each country were represented using penalized regression splines tuned by generalized cross validation. We also fitted a linear regression model to the data and compared models performance using AIC and an F-test (see the Supplementary Materials). As a further check against the possibility that there exist thresholds in these relationships, piecewise linear regression models were fitted to data for each country^61^.

The relationships between *per capita* GDP (constant 2010 USD) and polio incidence (averaged from 1980–2015) were evaluated by GAM and Analysis of Variance (ANOVA) after data were scaled and centered. We also divided the data into two groups: countries whose *per capita* GDP exceeds $\$\mathrm{1,000}$, and countries with GDP less than $\$\mathrm{1,000}$. We fitted a GAM to each group of data separately for further analysis. We were interested in assessing the possible threshold level of vaccine uptake and to visualize its association with the low income and high income countries. To do so, we determined univariate class intervals for the routine vaccine coverage data. The class intervals are defined using different styles. The best style is chosen based on the two indices: the goodness of variance fit measure (GVF), and the tabular accuracy index (TAI)^62^. The best method for our data with higher GVF and TAI values was the Jenks natural break. Jenks maximizes the similarity of numbers in groups by minimizing each class average deviation from the class mean, while maximizing each class deviation from the means of the other groups. The Jenks natural break provides a uniform interface to finding class intervals for continuous numerical variables^63^. A threshold level of 71% for mean vaccine uptake was estimated using the Jenks natural breaks classification method using the package ‘classInt’ in R.

### Ranking the Determinants of Polio Incidence

To quantify the relative importance of demographic and epidemiological factors in polio incidence, a set of explanatory characteristics was compiled for each country (see Supplementary Fig. S1). Candidate covariates were (i) population density, (ii) *per capita* unvaccinated births, (iii) percent rural and (iv) urban population growth, (v) percent forest and (vi) arable lands, (vii) percent of population with access to improved sanitation facilities, and (viii) *per capita* GDP obtained from the World Bank’s World Development Indicators^58^. To account for the possible climate predictors of transmission, (ix) average annual temperature, and (x) total annual precipitation were obtained for each country from the Centre for Environmental Data Analysis^64^. The availability of data restricted the analysis to 69 countries in the African, Eastern Mediterranean, Western Pacific, South-East Asia, Americas and European regions defined by WHO (see Supplementary Fig. S2). For each country, explanatory variables were centered and scaled for the period 1980–2015. To impute the missing data among predictors, first, the Pearson correlation coefficient was computed between each pair of predictors using all complete pairs of observations on those predictors for each country^65^. Next, a linear regression was fitted to the pair with highest correlation coefficient. The missing values were estimated based on the fitted model equation^66^.

To rank the predictors of polio incidence for each country, we used Random Forests, a machine learning algorithm that trains an ensemble of classification and regression trees and votes their collective predictions. For datasets with many input variables each containing a small sample size, a single tree classifier will have accuracy perhaps only slightly better than random. Combining trees grown using randomly selected features can improve accuracy by minimizing overfitting and producing unbiased estimates of modeling errors^60,67,68^. The model-free approach of machine learning algorithms like random forests enables higher predictive accuracy with assumptions concerning the relationships between predictor and response variables as well as data distribution^69^. In our study, taking into account our sample size and the number of predictors with high correlations among them, we used the algorithm implemented in the ‘randomForest’ package in R. In this implementation, each tree is constructed using a different bootstrap sample from the original data. About one-third of the data are left out of the bootstrap sample and not used in the construction of the tree. For each of the bootstrapped samples, the candidate predictors of polio incidence are randomly sampled, the variable providing the most information among samples is chosen at each node of a small decision tree, and the process is repeated^70^. Growing large numbers of trees does not overfit the data, and random predictor selection diminishes correlation among unpruned trees and keeps the bias low by taking an ensemble of unpruned trees^68^. To compute the generalization error, the data which are not in the bootstrap sample, called out-of-bag (OOB) data, are used at each iteration. We computed variable importance as the average increase in Mean Square Error (MSE) of predictions, $\Delta$ MSE, when OOB data for the variable are permuted while all others are left unchanged^67^. A zero or negative value of $\Delta$ MSE indicates the variable is not useful for prediction. For model training, the number of trees and the number of variables tried at each split were tuned independently for each country. We then compared the top three risk factors across countries to investigate geographic and epidemiological heterogeneity. In addition, a single random forests model was fitted to countries all-together as a check against possible idiosyncrasies of this approach. The coefficient of determination, ${R}^{2}$, was used as a goodness of fit. ${R}^{2}$ here is the square of Pearson correlation coefficent value between predicted and observed polio incidence. Also the percent variance explained by each model was reported.

In addition, to investigate the association between polio incidence and unvaccinated births, we fitted a generalized additive model to incidence data and *per capita* unvaccinated births. The residuals of the fitted GAMs were then used as a response variable in country-specific random forests models. This way the variance in incidence data that could not be explained by the *per capita* unvaccinated births, could potentially be described by other polio covariates (see Supplementary Fig. S3).

As a further out-of-sample test for model-data agreement, the fitted random forests models were confronted with incidence data from two endemic countries, Afghanistan and Pakistan. Three separate combinations of training and testing data were considered. First, we compared model output with the fitted data. Second, the data from 1980–2000 were used for training, with data from 2001–2014 reserved to test out-of-fit predictions. Finally, we explored one-step-ahead forecasts. Here, random forests models first were trained using the data from 1980–2000 and then tested with the incidence data in 2001. Next, the data from 2001 (one observation) were added to the training set (1980–2001) and the incidence of the next upcoming year (2002) was predicted. This process continued systematically until we had predicted polio incidence in all years up to our final year of data (2015). We checked the robustness of our modeling approach by comparing the performance of random forests with a parametric modeling approach, specifically linear regression.

### Polio Persistence

Epidemic fadeouts are known to result from a combination of factors, primarily the random sequences of infections in small populations (referred to as “demographic stochasticity”^26^) and the timescale of demographic processes, in particular the birth rate^27^. Thus, extinction risk is higher in small populations and/or those with low birth rates compared with large populations and/or high birth rates. However, recently, a novel contributory mechanism has been proposed: the metapopulation “rescue effect”^50^. This effect reflects the relative differences in spatial coupling, and hence potential recolonization, among highly connected (mainland) and isolated (island) communities. To characterize regional persistence of polio, we plotted the proportion of years with no reported cases of polio against the actual number of unvaccinated births (as defined above) averaged over 35 years (1980–2014). We contrasted polio persistence in islands with non-island settings. Island countries were identified according to Wikipedia (see the Supplementary Materials). Australia was regarded a non-island country. A generalized additive model with integrated smoothness estimation was fitted to these data. We also examined the local persistence of polio as a function of *per capita* unvaccinated births and fitted a segmented regression model to identify potential thresholds.

## Electronic supplementary material

Supplementary Material
