# Supplementary material for: Comparative epidemiology of poliovirus transmission
Source: Sci Rep. 2017 Dec 12;7:17362. doi: 10.1038/s41598-017-17749-5 (PMC5727041; doi:10.1038/s41598-017-17749-5)
Supplement: Supplementary file 1 — Supplementary Material [file 41598_2017_17749_MOESM1_ESM.pdf]

Title: Comparative epidemiology of poliovirus transmission

Authors: Navideh Noori[1,2], John M. Drake[1,2], Pejman Rohani[1,2,3]

[1] Odum School of Ecology, University of Georgia, Athens, GA, USA

[2] Center for the Ecology of Infectious Diseases, University of Georgia, Athens, GA, USA

[3] Department of Infectious Diseases, University of Georgia, Athens, GA, USA

Corresponding author: Navideh Noori, nnoori@uga.edu, 140 E Green St, Odum School of Ecology, University of Georgia, Athens, GA, USA, 30602. Phone: 334-332-7197.

Supplementary Material

Determinants of Polio Incidence. We investigated the importance of demographic, environmental, and socio-economic factors as key covariates of geographical variation in the patterns of polio transmission (Supplementary Fig. S1).

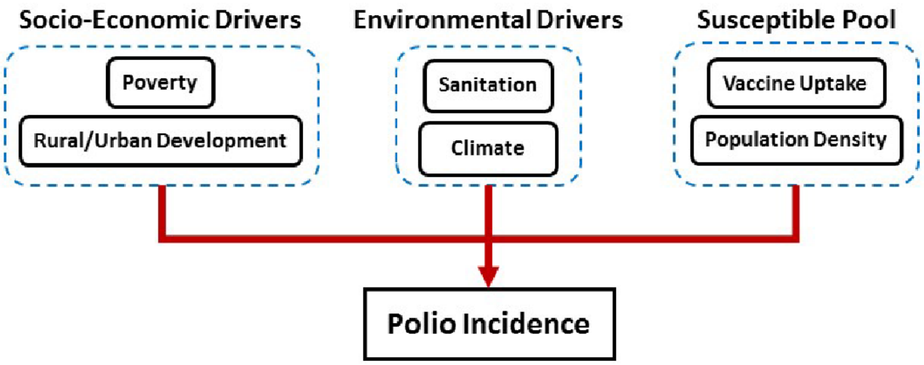

Supplementary Figure S1. Determinants of polio incidence pattern

The availability of adequate data restricted the analysis to 69 countries in the African, Eastern Mediterranean, Western Pacific, South-East Asia, Americas and European regions defined by WHO (Supplementary Fig. S2). The selected countries are Angola, Cameroon, Burkina Faso, Nigeria, Chad, Ethiopia, Madagascar, Mali, Central African Republic, Burundi, Côte d'Ivoire, Benin, Algeria, Guinea, Kenya, Mozambique, Ghana, Democratic Republic of the Congo, Malawi, Rwanda, Senegal, Sierra Leone, Togo, Tanzania, Uganda, Zambia, Zimbabwe, Lesotho, Mauritania, Peru, Afghanistan, Pakistan, Sudan, Egypt, Iraq, Oman, Iran, Saudi Arabia, Somalia, Syria, Yemen, Tunisia, Turkey, India, Bangladesh, Indonesia, Myanmar, Nepal, Thailand, China, Laos, Philippines, Papua New Guinea, Cambodia, Vietnam, Colombia, Dominican Republic, Guatemala, Haiti, Mexico, Bulgaria, Romania, Jordan, Botswana, Congo, Gambia, Guinea-Bissau, Sri Lanka.

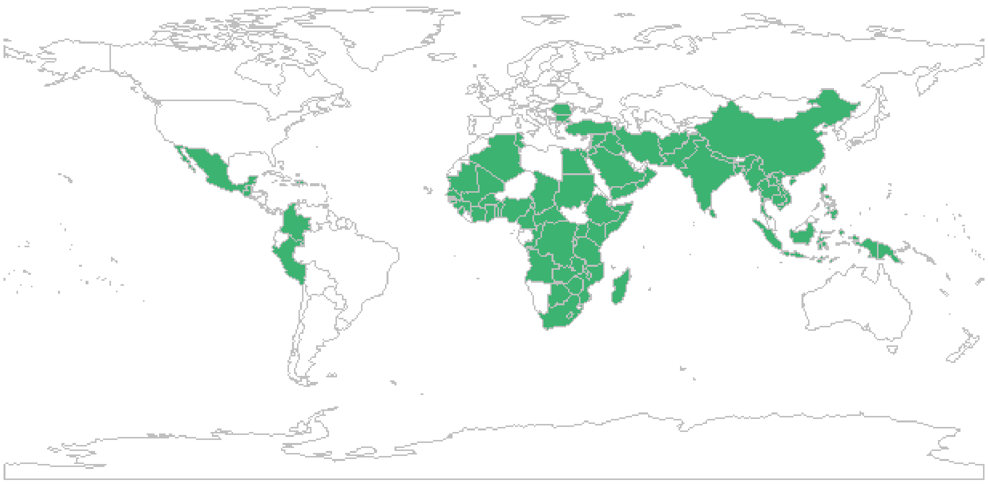

Supplementary Figure S2. 69 Countries considered in random forests methods. (R Core Team (2016)[1])

To assess the association between polio incidence and unvaccinated births, we fitted a generalized additive model (GAM) to incidence data and *per capita* unvaccinated births. The residuals of the fitted GAMs were then used as a response variable in country-specific random forests models. This way the variance in incidence data that could not be explained by the *per capita* unvaccinated births, could potentially be described by other polio covariates.

We ranked the covariates based on their relative importance in residuals of fitted GAMs by subtracting the R^2 of country-specific random forests models fitted to all predictors, R^2_{All}, from that of models in which predictors were fitted one at a time, R^2_i. The small value of R^2_{All} - R^2_i indicates higher predictive power of the factor i (Supplementary Fig. S3). Most of the variance in polio incidence was explained by population density, percent of people with access to improved sanitation facilities and *per capita* GDP (Supplementary Fig. S3).

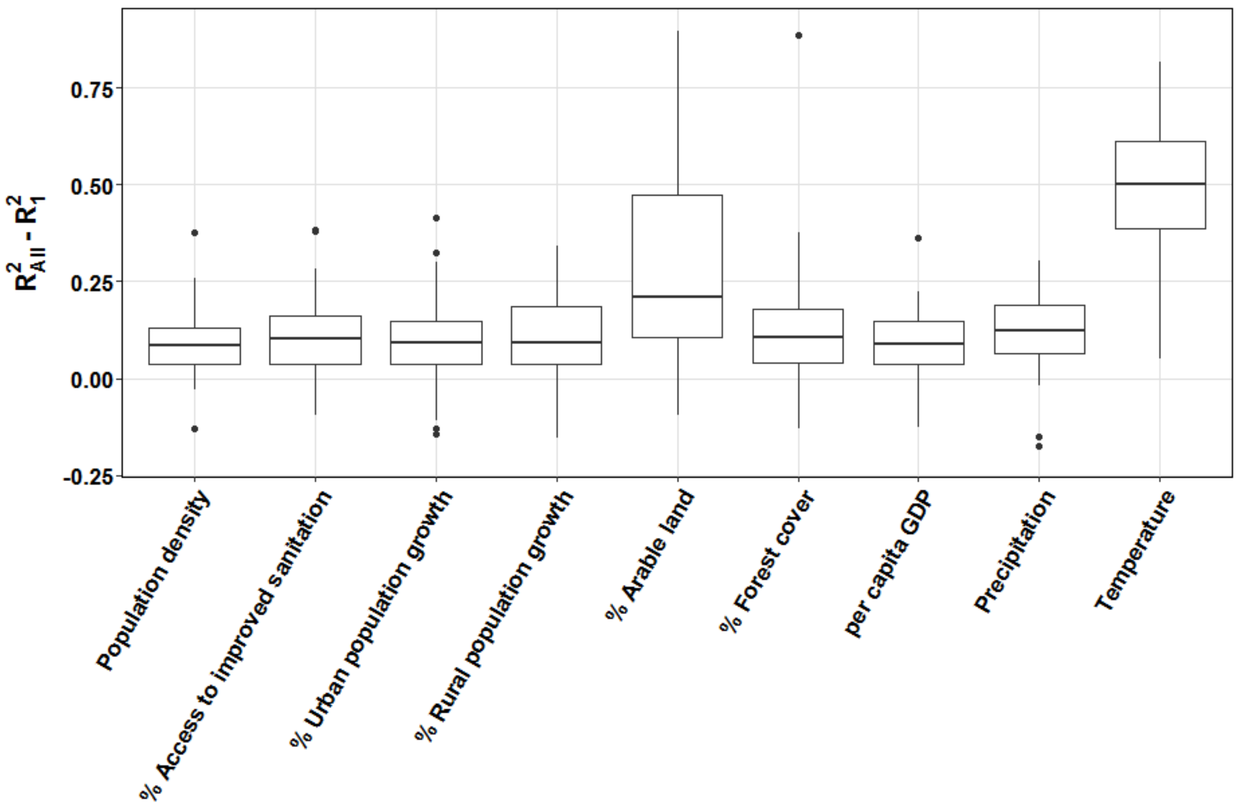

Supplementary Figure S3. Difference in R^2 of random forest models developed using 1) all predictors and 2) only one predictor. The response variable here is the residuals of (incidence rate ~ *per capita* unvaccinated births).

To explore the association between incidence, and *per capita* unvaccinated births in each country, we fitted a generalized additive model (GAM) with integrated smoothness estimation to the data. We used a GAM because of the flexibility it provides and the agnosticism regarding the response function (eg, linear, quadratic, etc.). To check the sensitivity of our conclusions to this choice, we also fitted a linear regression model to the incidence and the susceptible birth rate and compared model performance using AIC and an F-test. As shown in Fig. S4, for six randomly selected countries, GAM provides a better or similar fit to the data and is associated with lower AIC values

and P-value of less than 0.05 (based on the F-test). Therefore, we feel reassured that our application of GAM in our analyses is warranted.

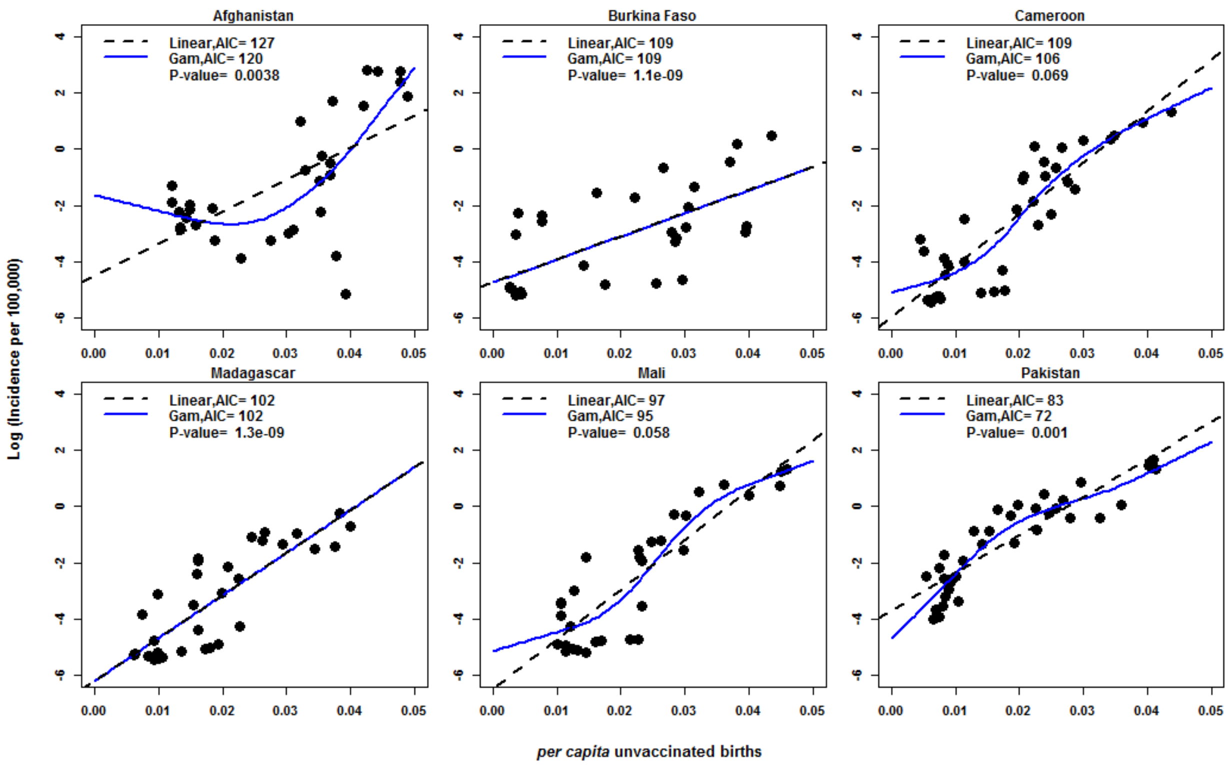

Supplementary Figure S4. Plots of logarithmic incidence rate against *per capita* unvaccinated births for six different countries. Linear Model (LM) and Generalized Additive Model (GAM) were fitted to each country data points. Akaike information criterion (AIC) values of the fitted models are given.

To identify potential thresholds in the association between incidence, and *per capita* unvaccinated births in each country, piecewise (segmented) linear regression models were fitted to data[2] using the 'segmented' package in *R*[2]. Segmented regression estimates a new model having broken-line relationships with the predictor. This relationship is defined by the slope parameters and the break-points. Results show a threshold in *per capita* unvaccinated births through fitted segmented

models, below which there is negligible change in polio incidence, however this threshold was not detected in all countries. (Supplementary Fig. S5).

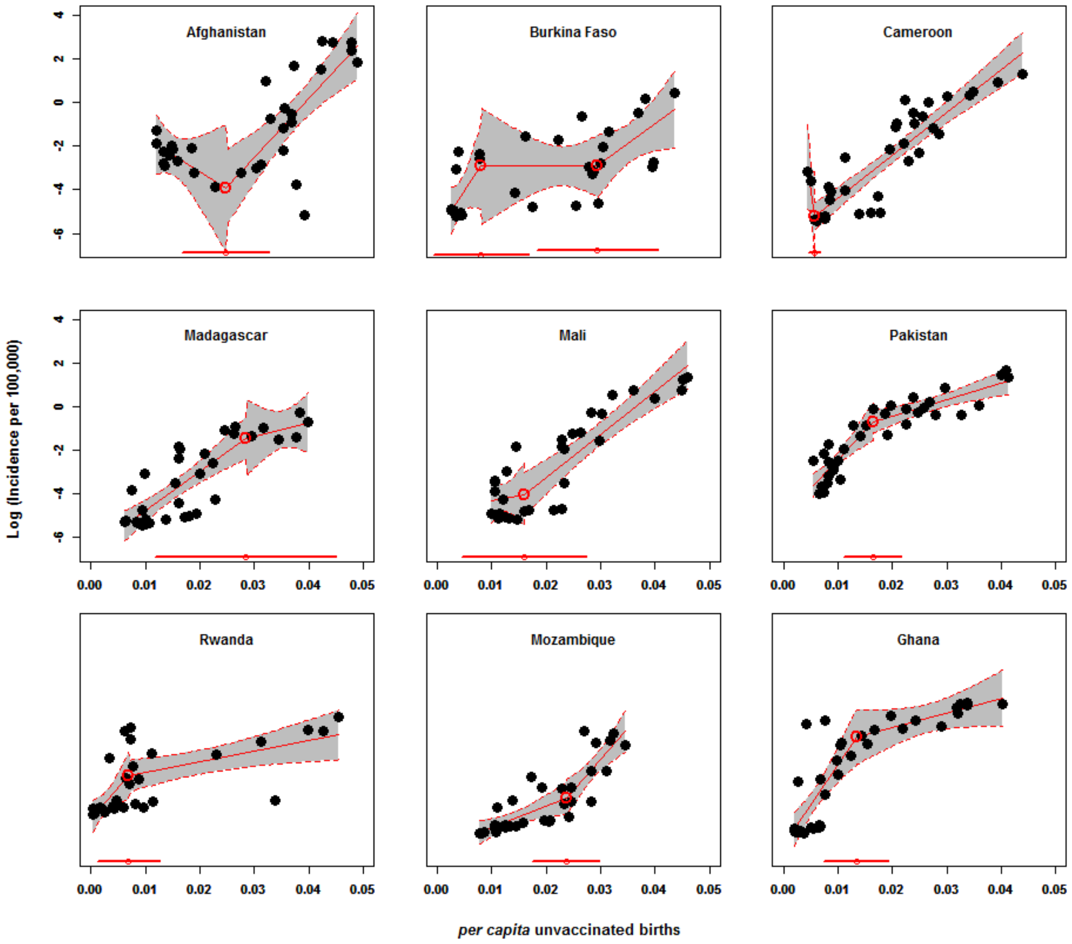

Supplementary Figure S5. Plots of logarithmic incidence against *per capita* unvaccinated births for nine different countries. Shaded area shows the confidence interval of the fitted segmented model. Red circle and red line at the x axis show the breakpoint and the confidence interval of the point, respectively.

L-shaped plot of country-specific *per capita* GDP against polio incidence (Fig. 3), tell us there are two types of countries, (i) low income countries with high incidence and (ii) high income countries with low incidence. Thus, we divided the data to two groups: countries whose GDP exceeds

$1,000, and countries with *per capita* GDP < $1,000. We explored the impact of economic growth on polio incidence for each group separately by fitting a general additive model (GAM) (See Supplementary Table S1). Among high income countries, there was a significant reduction in model deviance using the smoothed response to vaccination (P-value =0.002), however the smoothed response to *per capita* GDP was not statistically significant. Also for high income countries the smoothed response to the interaction between GDP and vaccine uptake was significant (P-value=0.022) (See Supplementary Table S1). Among countries whose GDP is less than $1000, only the smoothed response to vaccination was statistically significant (P-value =0.048) (Supplementary Fig. S6(b)).

Supplementary Table S1. Fitted GAM to two groups of data: countries whose GDP exceeds $1,000, and countries with *per capita* GDP < $1,000

	***per capita* GDP < $1000**				***per capita* GDP >$1000**			
	**edf[a]**	**Ref.df[a]**	**F[a]**	**p-value**	**Edf**	**Ref.df**	**F**	**p-value**
GDP	1	1	0.115	0.736	1	1	0.202	0.46
Vaccination	1	1	4.19	0.048	2.51	2.89	7.14	0.002
GDP * Vaccination	1	1	0.41	0.525	13.74	77	0.301	0.022

[a]edf: estimated degree of freedom, Ref.df: degrees of freedom for reference distributions, F: a test of whether the smoothed function significantly reduces model deviance. Two-sided p-values < 0.05 indicate significant relationships.

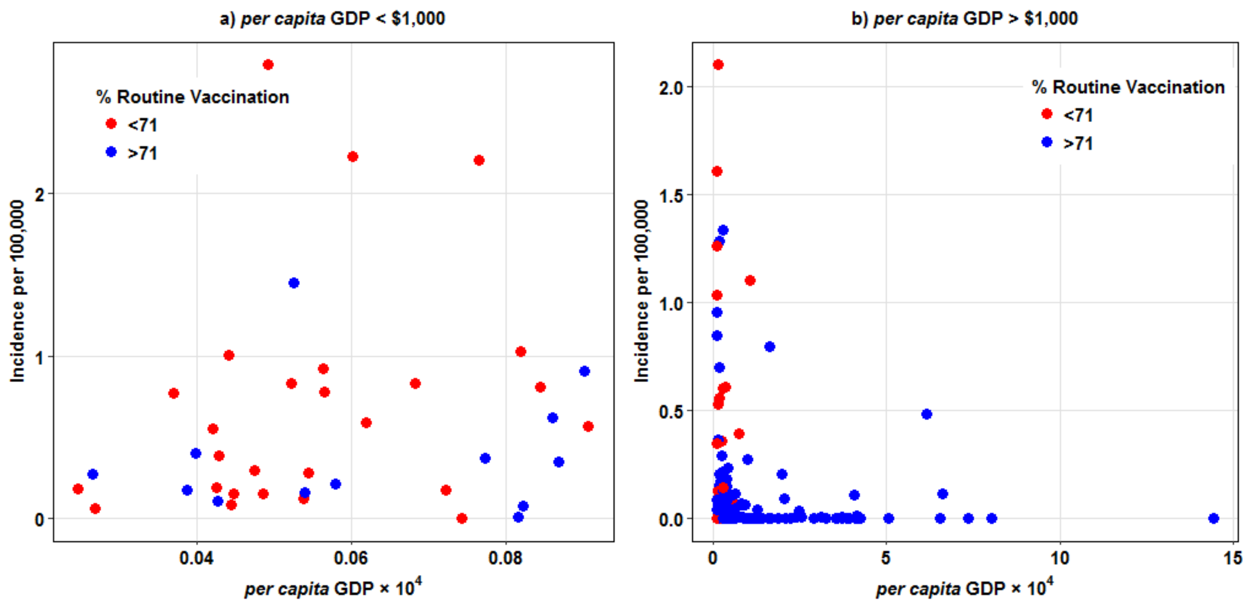

Supplementary Figure S6. Polio incidence and *per capita* GDP (constant 2010 US$) over the period 1980-2015 for countries with a) *per capita* GDP < $1,000 and b) *per capita* GDP > $1,000. Blue color points indicate countries with the vaccination coverage > 71% and red color points indicate countries with the vaccination coverage < 71%.

A single random forests model was fitted to countries all-together as a check against possible idiosyncrasies of this approach. Population density, percent of people with access to improved sanitation facilities and *per capita* GDP were the most predictive covariates of polio incidence globally. The negative values of R^2_{All} - R^2_i mean the model fitted to one predictor performs better than the model in which all predictors were fitted (See Supplementary Fig. S7 and Table S2). However as shown in Table S2, the fitted model to all predictor explain 21 percent of variance in the data. These results indicates the geographic heterogeneity in polio incidence. To account for this variance and to identify the role of other covariates, a separate model needs to be fitted to each country (Tables 2 & Fig. 4).

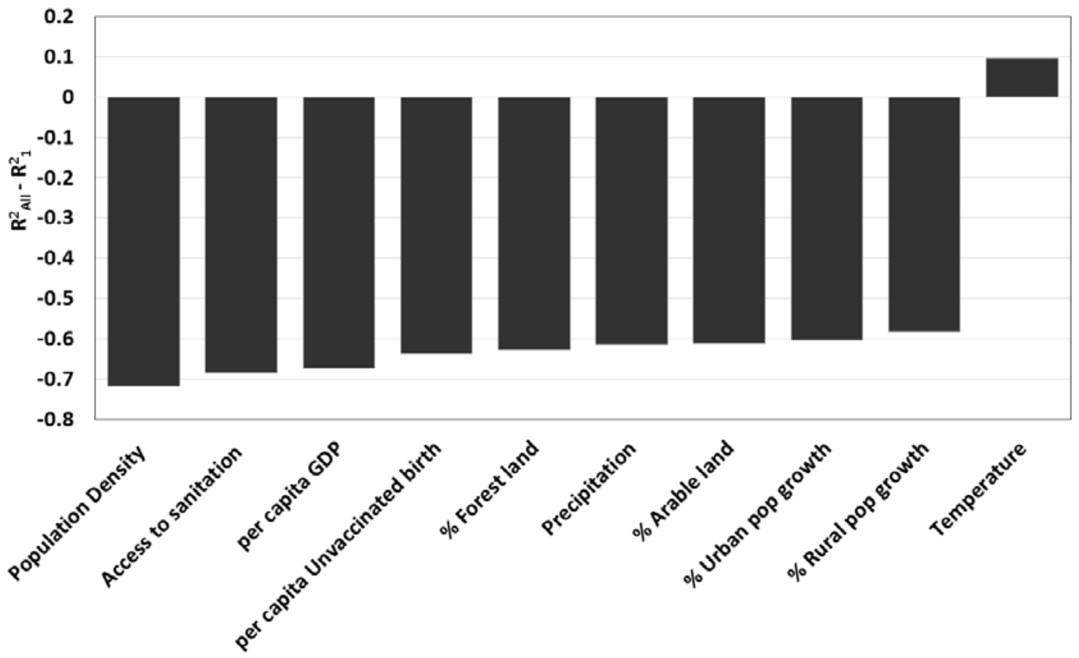

Supplementary Figure S7. Difference in R^2 of single random forests model developed for countries all-together using 1) all predictors and 2) only one predictor at a time.

Table S2. ΔMSE values for each predictor variable based on the single random forests model developed for countries all-together.

% of population with access to Improved sanitation facilities	% Urban population growth	% Rural population growth	% Forest cover	% Arable land	Population density	*per capita* GDP	Total precipitation	Mean temperature	*Per capita* unvaccinated births	Variance Explained	R^2
5.76	5.05	6.42	5.68	5.90	5.42	8.79	6.14	5.59	7.56	0.21	0.16

Random Forest

We ranked the covariates of polio incidence using the country-specific random forests models. The average increase in Mean Square Error (MSE) of predictions, ΔMSE, was considered as the variable importance index (See Supplementary Table S3). To identify any possible grouping among predictors, the Pearson correlation matrix of covariates rankings was calculated. Circles mark significant correlations ($P<0.05$) (Supplementary Fig. S8).

We found out that the ranking of percentage of urban population growth is negatively correlated with the ranking of temperature among 69 countries ($r=-0.37$). This shows that in countries which urban population growth is an important predictive covariate of polio incidence, temperature or vice versa. The same interpretation applies to percent rural population growth versus *per capita* GDP or versus percent forest cover with $r=-0.24$ and -0.3 respectively. No other significant correlation was identified among predictive power of covariates. This indicates that there are many statistically significant covariates of change in polio incidence rather than a universal pattern.

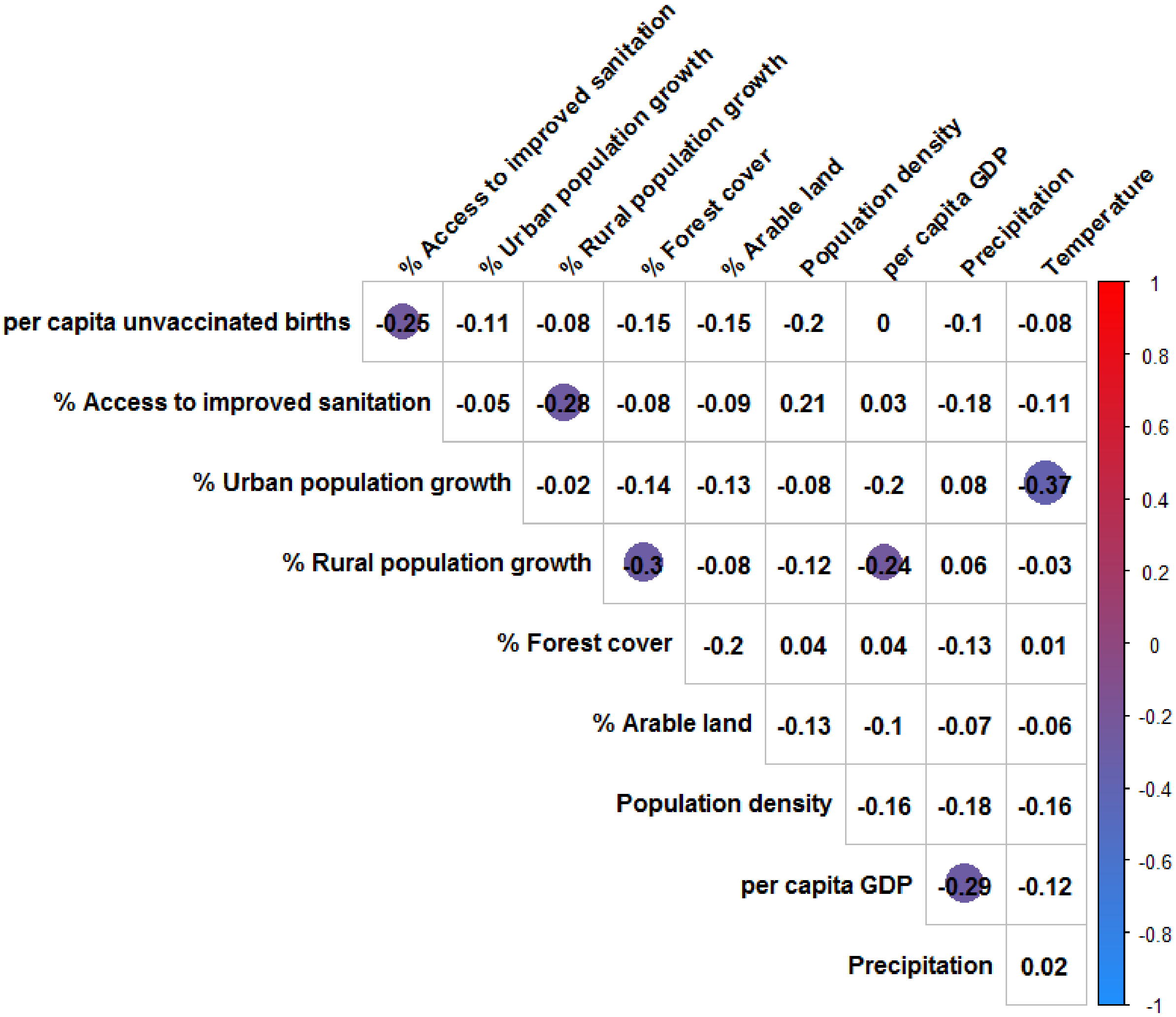

Supplementary Figure S8. Pearson correlation matrix of predictors ranking based on the ΔMSE values of random forests analysis. Cells with no circle indicate the nonsignificant correlation (P>0.05). The circle size increases with the correlation size.

Table S3. ΔMSE values for each predictor variable and each country based on the random forests analysis.

Region	Country	% of population with access to Improved sanitation facilities	% Urban population growth	% Rural population growth	% Forest cover	% Arable land	Population density	*per capita* GDP	Total precipitation	Mean temperature	*Per capita* unvaccinated births	Variance Explained	R^2
African	GIN	3.44	2.64	2.37	3.61	3.25	3.30	2.52	1.79	2.70	2.65	0.40	0.85
	CMR	5.20	3.86	0.82	3.80	3.11	5.58	4.69	-1.10	-0.96	3.12	0.83	0.96
	BFA	9.81	3.80	4.40	5.60	7.69	8.42	5.30	2.03	0.77	2.03	0.94	0.99
	NGA	5.93	5.58	6.49	6.39	6.30	6.73	3.49	3.23	1.08	3.26	0.31	0.86
	TCD	16.97	5.21	8.72	16.16	15.19	16.13	20.57	9.78	10.87	12.38	0.08	0.84
	ETH	4.17	0.97	2.38	5.01	2.56	4.32	2.11	-0.42	3.68	2.05	0.49	0.89
	MDG	4.70	2.59	1.37	4.37	4.00	3.89	2.99	2.01	1.22	5.30	0.77	0.95
	MLI	2.26	-0.33	2.64	3.10	1.82	3.94	1.70	3.19	0.56	5.58	0.69	0.95
	CAF	7.33	9.75	2.48	9.80	-0.55	6.86	8.16	4.93	4.06	5.90	0.57	0.92
	BDI	17.85	9.04	3.00	-0.51	5.45	20.13	3.29	-0.13	11.66	16.81	0.52	0.88
	CIV	2.92	3.45	3.13	2.40	0.59	3.11	1.51	-1.46	-1.58	2.59	-0.01	0.80
	COD	5.14	5.08	1.48	5.43	4.87	6.20	3.31	a		4.30	0.18	0.82
	GHA	5.24	5.49	9.71	5.66	5.96	5.96	3.29	1.04	9.27	12.53	0.78	0.96
	BEN	7.16	5.21	4.64	12.45	6.65	6.71	8.09	4.31	5.25	3.07	0.78	0.95
	DZA	3.67	4.08	3.41	4.03	3.67	4.13	-1.67	1.32	-1.46	3.32	0.87	0.97
	AGO	4.54	-0.59	-0.24	4.50	1.97	3.41	0.66	-1.03	4.15	3.77	-0.16	0.76
	KEN	4.09	2.09	3.83	1.78	2.36	4.60	2.14	-0.60	0.77	3.03	0.51	0.95
	MOZ	10.26	9.31	4.30	6.48	9.28	10.38	4.56	1.64	2.81	3.03	0.81	0.96
	MWI	9.88	-0.98	-2.20	13.64	3.47	12.73	1.43	3.45	12.53	14.75	0.70	0.94
	RWA	0.88	3.18	8.69	7.15	5.35	7.04	6.33	0.21	6.59	4.60	0.55	0.91
	SEN	5.28	1.27	0.45	7.14	0.41	5.44	-0.88	-0.96	1.20	2.43	0.71	0.94
	SLE	1.32	2.44	1.59	1.81	2.63	1.64	1.91	0.83	1.06	3.46	0.17	0.86
	TGO	7.65	5.60	4.64	12.33	8.87	14.88	4.37	-0.97	-1.88	3.28	0.77	0.96
	GMB	0.75	-1.13	0.43	1.47	-0.35	1.37	1.94	0.00	1.22	1.11	-0.08	0.82
	UGA	10.46	2.84	-0.46	12.47	9.59	11.05	11.32	-2.47	-3.25	12.14	0.07	0.82
	ZMB	2.55	2.30	-0.06	12.53	9.27	13.58	2.87	0.75	2.75	2.55	0.58	0.92
	ZWE	2.68	0.58	2.40	1.60	4.18	2.90	0.74	1.42	5.22	1.36	0.41	0.91
	TZA	4.88	3.17	2.02	3.25	2.16	5.23	4.63	1.32	1.30	1.23	0.38	0.92
	MRT	2.74	4.90	4.14	2.22	3.26	2.67	-1.73	4.66	-1.29	2.58	0.30	0.86
	BWA	2.36	8.15	0.56	2.28	1.05	3.08	3.08	0.26	2.39	1.53	0.66	0.92
	COG	7.82	5.67	7.29	6.52	8.64	6.77	5.95	3.59	-1.09	-2.98	-0.12	0.91

	LSO	3.63	2.28	3.80	3.53	0.58	4.00	2.88	0.59	0.68	3.29	0.77	0.96
	GNB	5.57	4.48	-0.07	5.10	-1.37	5.89	0.45	-1.13	-0.32	4.10	0.05	0.74
	ZAF	3.80	2.48	2.79		1.77	3.48	2.04	-0.84	1.88	1.61	0.30	0.78
	PER	2.00	4.01	2.54	4.38	2.80	3.95	-1.00	0.68	-1.74	5.38	0.92	0.98
	COL	4.04	3.04	0.64	2.95	1.20	0.50	1.36	-0.81	1.25	3.13	0.38	0.77
Americas	DOM	6.82	5.17	-0.92	7.12	1.49	7.20	-0.25	-0.54	-1.04	2.62	0.53	0.89
	GTM	3.36	1.33	2.76	3.88	0.00	3.81	1.21	-0.19	-1.01	1.51	0.35	0.83
	HTI	7.45	5.37	4.06	6.11	2.14	7.33	5.48	0.70	1.46	5.89	0.45	0.83
	MEX	18.86	16.15	0.37	18.06	16.91	18.16	-2.97	-6.51	3.96	1.22	0.38	0.95
	AFG	3.95	8.87	9.68		1.73	0.33	1.02	-1.54	-1.58	8.63	0.62	0.92
	PAK	13.81	15.95	13.48	23.10	5.53	13.12	18.55	11.21	0.12	21.18	0.72	0.95
	SDN	1.16	1.79	-0.06	1.27	1.61	0.25	3.59	0.20	-2.73	-1.52	-0.17	0.92
	EGY	21.33	3.78	10.49	21.10	6.80	21.60	21.59	4.40	0.74	26.27	0.90	0.97
	IRQ	8.26	7.44	2.73	8.52	5.84	8.61	2.48	1.70	-0.06	6.26	0.56	0.95
	OMN	8.67	10.97	-1.69		8.82	13.08	12.14	-3.81	3.89	13.27	0.34	0.87
Eastern Mediterranean	IRN	6.32	2.69	1.67	2.87	3.41	6.90	2.12	1.75	1.98	2.40	0.67	0.93
	SAU	5.43	9.11	1.33		6.45	6.24	4.99	-0.99	2.88	5.90	0.76	0.95
	SOM	2.72	6.00	3.53	2.98	-2.93	4.08		-1.11	-2.56	-0.69	-0.34	0.79
	SYR	2.32	1.01	2.04	1.94	3.88	3.25		1.70	1.19	6.63	0.91	0.98
	YEM	13.27	11.13	10.91		6.95	13.01	9.48	8.71	9.31	11.08	0.59	0.89
	TUN	3.65	3.32	0.25	1.99	2.28	2.29	0.61	-1.41	-0.24	3.54	0.30	0.86
	JOR	0.41	0.36	1.79	-0.02	-0.78	-0.28	-1.50	3.50	2.35	-0.36	-0.01	0.89
	TUR	6.98	4.59	3.21	7.31	2.83	8.12	8.81	-1.88	-2.33	4.49	0.87	0.97
Europe	BGR	1.14	0.80	1.12	1.33	-1.09	2.18	-0.92	0.19	0.47	-0.52	-0.14	0.90
	ROU	2.51	4.29	2.26	0.16	3.74	2.47	1.74	1.10	-0.03	2.28	0.43	0.95
	IND	1.29	5.78	5.32	2.75	2.70	3.22	2.74	-1.86	0.30	4.96	0.85	0.96
	BGD	3.51	-0.39	5.90	1.40	9.92	2.45	1.67	-0.54	-0.01	0.92	0.63	0.94
	IDN	3.25	-2.01	1.60	2.03	3.17	2.46	3.63	-0.54	0.76	1.19	0.23	0.89
South-East Asia	MMR	22.02	7.24	15.97	15.62	8.62	22.65	12.19	-1.54	0.64	10.65	0.94	0.98
	NPL	4.19	3.60	1.79	2.98	3.45	4.61	4.27	2.27	-0.13	4.84	0.60	0.88
	THA	4.35	1.43	3.33	3.30	1.48	3.98	3.85	-1.40	2.05	4.60	0.85	0.96
	LKA	2.18	2.34	3.72	3.43	2.66	2.64	2.14	-0.87	2.13	2.57	0.66	0.98
	CHN	6.83	9.68	6.37	6.34	4.47	4.96	5.34	-2.08	-0.55	2.99	0.81	0.97
	LAO	0.30	-1.07	4.62	1.01	1.56	0.28	1.82	-0.99	1.58	4.28	0.18	0.94
Western Pacific	PHL	11.69	6.40	4.91	-2.22	1.27	12.88	0.18	-1.90	-2.42	3.81	0.87	0.97
	PNG	1.05	11.80	-0.89	10.52	3.39	4.64	-4.23	2.97	-1.29	0.28	0.53	0.91
	KHM	4.51	2.93	-1.08	4.39	3.70	3.24	3.78	-0.30	2.57	3.29	0.73	0.94
	VNM	4.09	2.78	3.77	4.07	2.51	4.66	3.15	-1.53	1.10	4.24	0.78	0.96

[a] Blank cells indicate variables with no available data.

Model validation and polio forecasting. We examined the reliability of random forests forecasting results by additionally fitting linear regression models to the incidence data from two of the remaining endemic countries, Afghanistan and Pakistan (Supplementary Fig. S9). Three training and testing sets were considered: (i) fitted data, (ii) out-of-fit predictions, and (iii) one-step-ahead predictions. More explanations were given in the methodology section of the main text. For the fitted data, linear regression models with R^2=0.74, 0.78, 0.33, and 0.91 respectively for Afghanistan, Pakistan, Nigeria and India, had a fairly acceptable prediction accuracy. For the out-of-fit and one-step-ahead predictions, linear regression models performed poorly and often predicted negative values of incidence (Supplementary Fig. S9).

Afghanistan

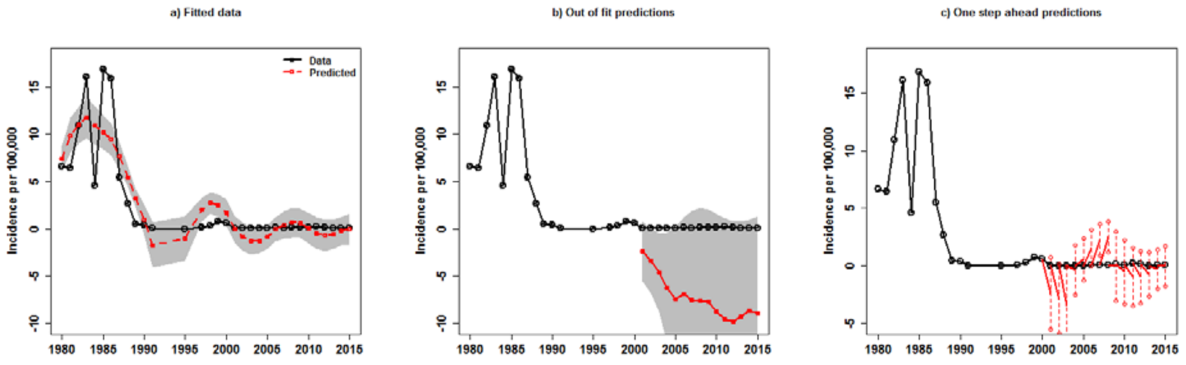

Pakistan

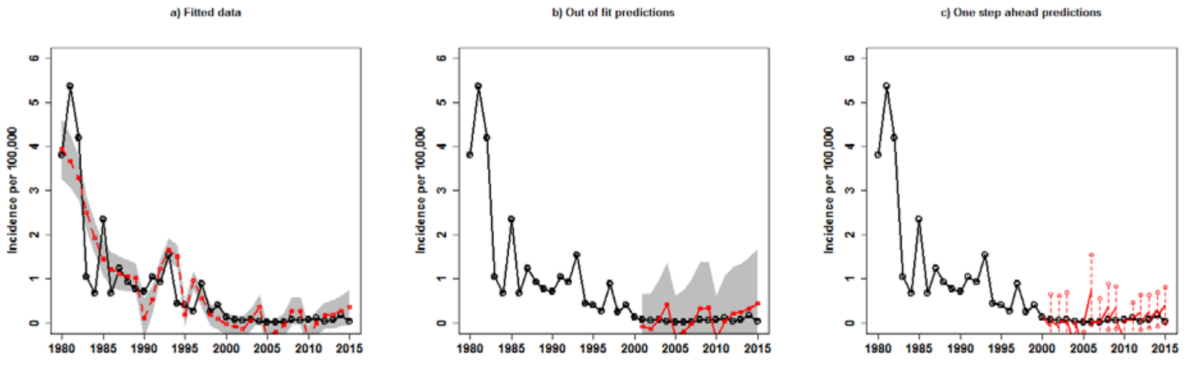

Nigeria

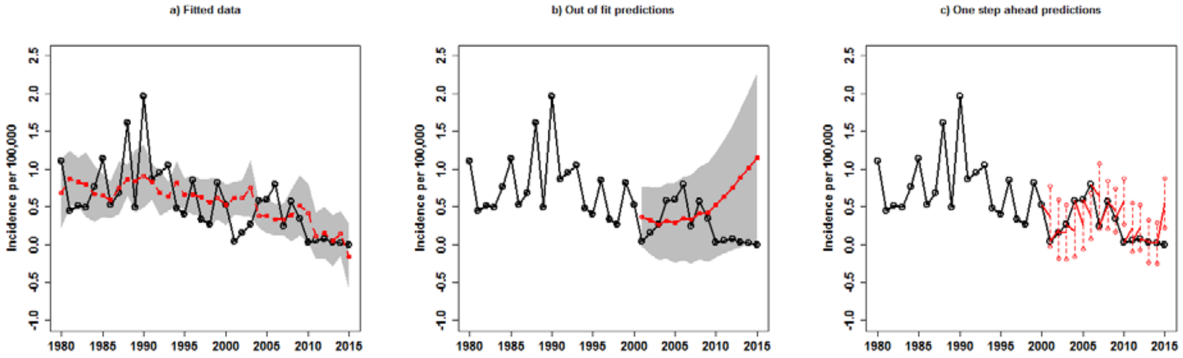

India

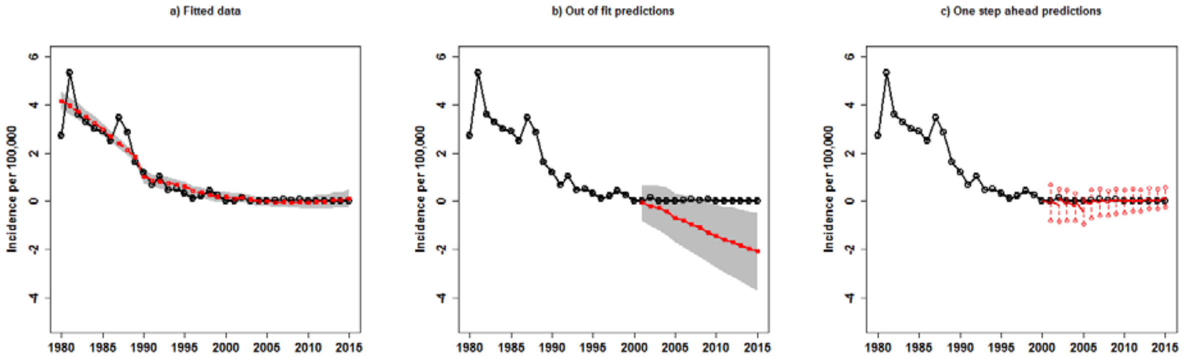

Supplementary Figure S9. Comparison of predicted polio incidence by linear regression models versus observed values for Afghanistan, Pakistan, Nigeria and India using three training and testing sets a) fitted data, b) out-of fit-predictions, c) one-step-ahead predictions.

Polio persistence. Regional persistence of polio was evaluated in island and non-island countries as a function of number of unvaccinated births as well as *per capita* unvaccinated births. Segmented model was fitted to the data. The segmented regression model showed a threshold in the extinction frequency of polio for both island and non-island countries (Supplementary Fig. S10). Below this breakpoint, polio incidence is unable to remain endemic in the community. Selected island countries are Antigua and Barbuda, Bahrain, Bahamas, Barbados, Brunei Darussalam, Comoros, Cabo Verde, Cuba, Cyprus, Dominica, Dominican Republic, Fiji,

Micronesia, United Kingdom of Great Britain and Northern Ireland, Haiti, Grenada, Indonesia, Ireland, Iceland, Jamaica, Japan, Kiribati, Saint Kitts and Nevis, Saint Lucia, Sri Lanka, Madagascar, Maldives, Marshall Islands, Malta, Mauritius, New Zealand, Philippines, Palau, Papua New Guinea, Singapore, Sao Tome and Principe, Solomon Islands, Seychelles, Timor-Leste, Tonga, Trinidad and Tobago, Tuvalu, Saint Vincent and the Grenadines, Vanuatu, Samoa. Australia was regarded a non-island country.

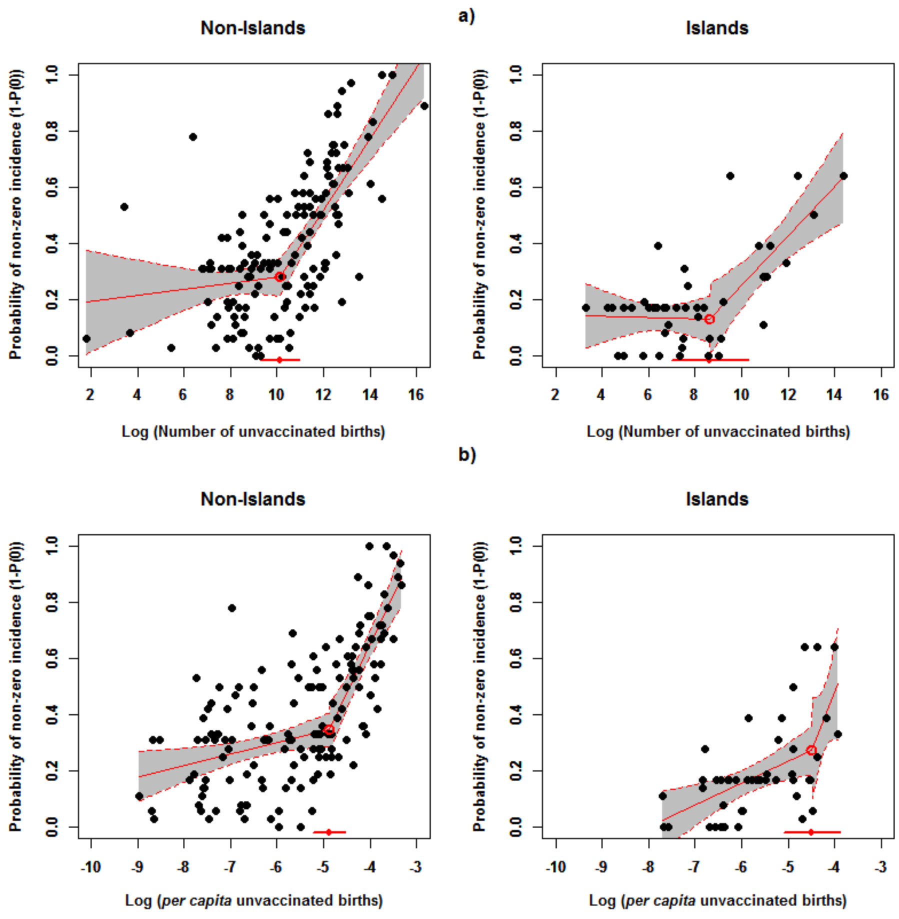

Supplementary Figure S10. Persistence of polio in island and non-island countries. Y axis is proportion of years in which cases of polio were non-zero for the period 1980-2015. X axis is a) number of unvaccinated births and b) *per capita* unvaccinated births. Shaded area for the fitted segmented models shows the confidence intervals. Red circle and red line at the x axis show the breakpoint and the confidence interval of the point, respectively.

One of the top ranked predictors of polio incidence in our study was percent forest cover. For further interpretation, we plotted the incidence data versus percent forest cover of randomly selected countries (Supplementary Fig. S11). Surprisingly, percent forest cover had a positive relationship with polio incidence. In these countries, by increasing the percent forest cover, population density and percent of people with access to improved sanitation facilities decreased. More explanation is given in discussion section of the main text.

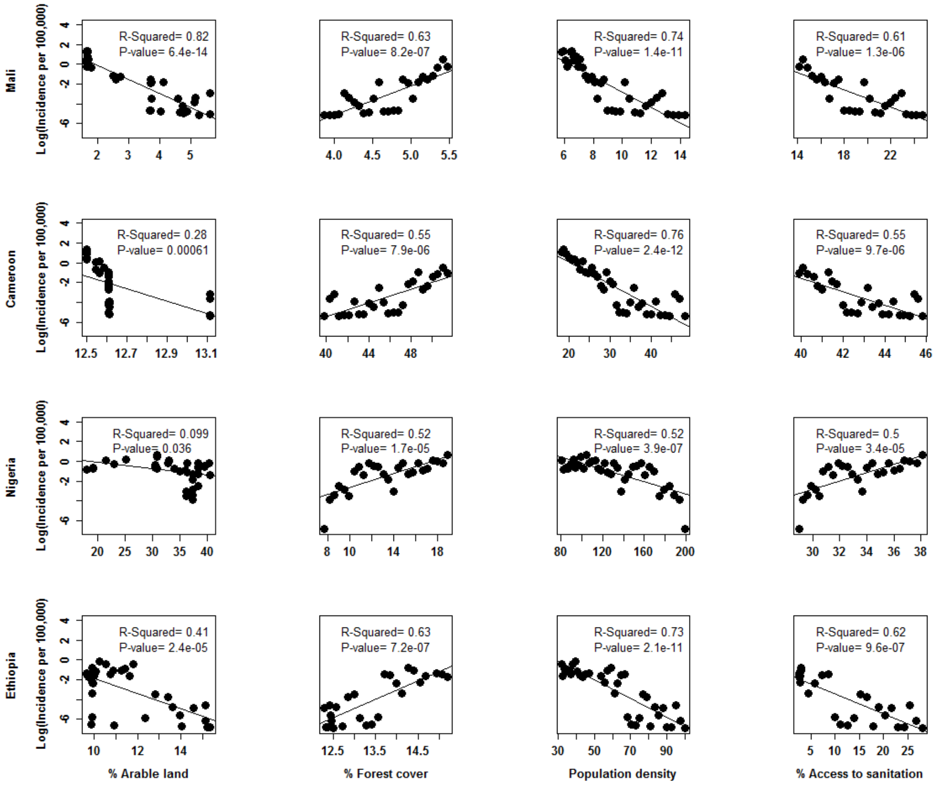

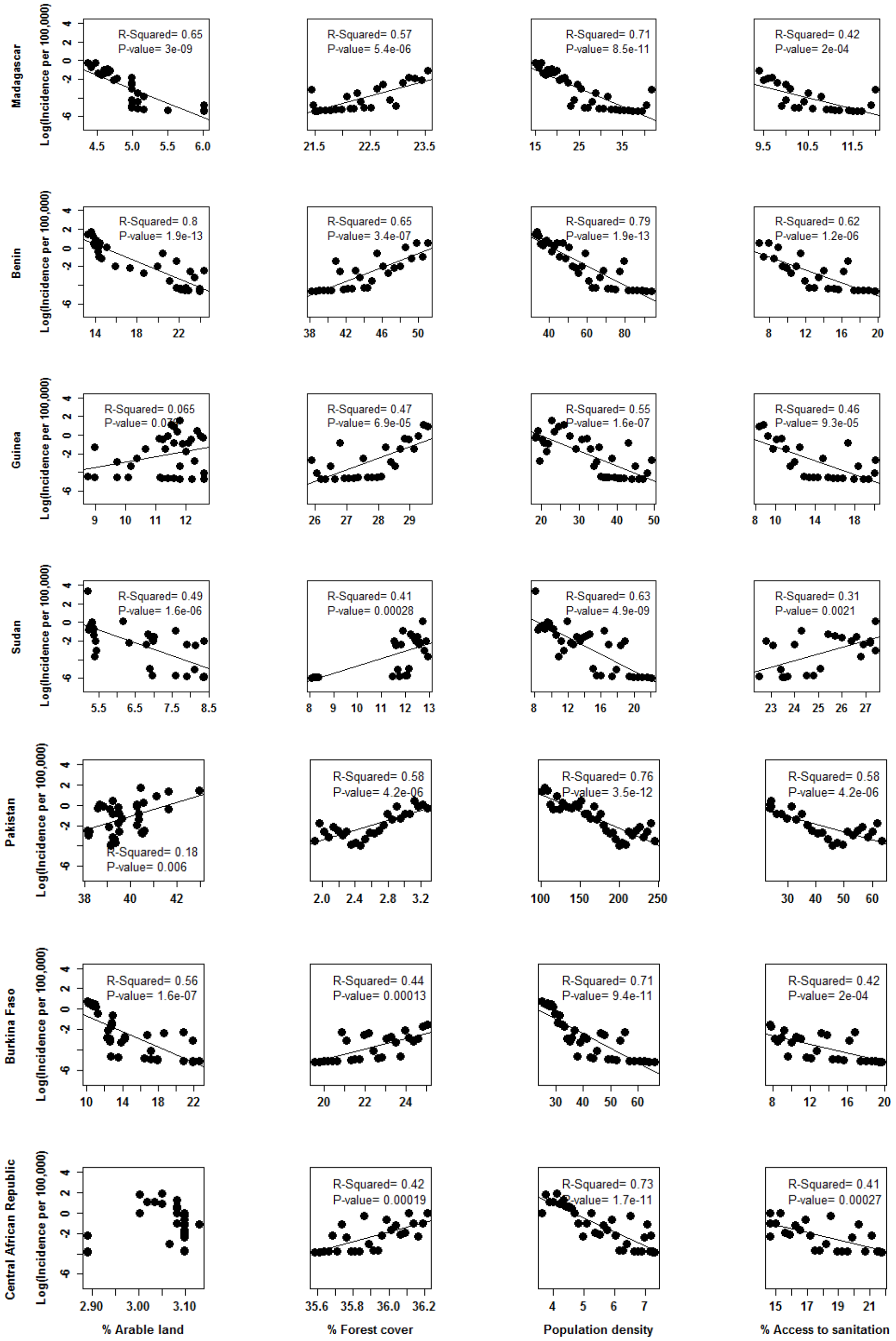

Supplementary Figure S11. Plots of logarithmic incidence rate against percent arable and forest lands, population density, and percent of people with access to improved sanitation facilities for 11 different countries. The R-squared and p-value of fitted linear regression model were given for each country.

References

1. R Core Team. R: A language and environment for statistical computing. R Foundation for Statistical Computing, Vienna, Austria. URL https://www.R-project.org/. (2016)

2. Muggeo VMR. Estimating regression models with unknown break-points. *Stat Med*. **22,** 3055-3071 (2003).
